# The Role of Scalp EEG Recordings During Cortical Visual Prosthesis Testing

**DOI:** 10.1111/aor.15023

**Published:** 2025-05-27

**Authors:** Katarina Stephan, Philip R. Troyk, Gislin Dagnelie, Stuart Cogan, Michael P. Barry, Patricia Grant, Frank Lane, Janet P. Szlyk, Maggie McNulty, Wim van Drongelen, Tuan H. Pham, Kelsey L. Stipp, Meesa Royster, Vernon L. Towle

**Affiliations:** ^1^ Stritch School of Medicine Loyola University Chicago Maywood Illinois USA; ^2^ Pritzker Institute of Biomedical Science and Engineering Illinois Institute of Technology Chicago Illinois USA; ^3^ Wilmer Eye Institute Johns Hopkins University Baltimore Maryland USA; ^4^ Department of Bioengineering The University of Texas at Dallas Richardson Texas USA; ^5^ The Chicago Lighthouse Chicago Illinois USA; ^6^ Department of Ophthalmology The University of Illinois at Chicago Chicago Illinois USA; ^7^ Department of Psychology Illinois Institute of Technology Chicago Illinois USA; ^8^ Department of Neurology Rush University Medical Center Chicago Illinois USA; ^9^ Department of Pediatrics The University of Chicago Chicago Illinois USA; ^10^ Department of Neurology The University of Chicago Chicago Illinois USA; ^11^ Department of Neuroscience Brown University Providence Rhode Island USA

**Keywords:** direct stimulation of human visual cortex, intracortical human visual prosthesis, minimizing the risk of seizures during direct intracortical stimulation, phosphenes from direct cortical stimulation, scalp EEG recordings during intracortical prosthesis testing, visual prosthesis

## Abstract

**Introduction:**

This article highlights the value of recording scalp electroencephalogram (EEG) from two individuals with blindness who received intracortical visual stimulators to create artificial vision. Given the known risk of cortical stimulation inducing seizures, we recorded occipital scalp EEG as a safety precaution. Over a 3‐year period, over 330 h of EEG data were collected.

**Methods:**

Twenty‐five wireless floating microelectrode arrays (WFMAs), each containing 16 stimulating electrodes, were implanted in the right dorsolateral occipital cortex of the first participant, totaling 400 independently controlled stimulating electrodes. Similarly, 32 WFMAs were implanted in the second participant's cortex, totaling 512 electrodes. Phosphenes were characterized and mapped to align their locations in the visual field to create camera‐driven imagery of the visual environment.

**Results:**

Scalp EEG recordings during intracortical stimulation provided early warning of impending epileptic activity, reducing the risk of stimulation‐induced seizures. In two instances, seizures occurred during direct cortical stimulation and were visible in the scalp EEG recordings. Normal electrically evoked potentials (eEPs) were also evident in the EEG records.

**Conclusion:**

Scalp EEG can be valuable to alert researchers to impending seizures. However, it is not required for conditions in which low levels of stimulation are employed.

**Trial Registration:** Clinical Trial Number NCT04634383

## Graphical abstract

This article describes the role of recording scalp EEG for projects that are developing cortical visual prosthesis systems. It concludes that EEG is not required during basic threshold and spatial mapping studies, but should be considered when higher levels of stimulation and video‐camera stimulation are employed.

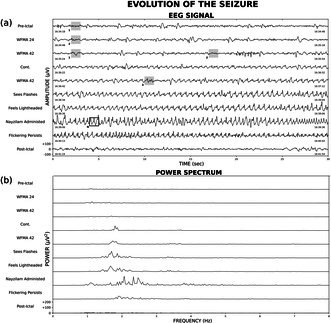

## 1 Introduction

Early reports preparing for prosthetic implants in animals assessed safe levels of stimulation for various types of electrodes [1, 2, 3, 4]. Our group interviewed 12 out of the 16 individuals, or their families, who received visual cortical implants, three of whom reported experiencing generalized seizures as a result of cortical stimulation [5]. Among the 40 known humans with cortical implants in the medial or lateral occipital cortex [5, 6, 7, 8, 9, 10, 11, 12, 13, 14], eight (20%) experienced stimulation‐induced seizures, one of which was fatal [5]. Although the death of neurons and mortality due to seizures have been widely discussed [15], we are aware of only four studies that have recorded EEG during [5] electrical stimulation of the human visual system [12, 13, 14]. The historic seizure rate suggests that some form of protection must be afforded to subjects undergoing artificial cortical stimulation. Because our implants cannot record intracranial EEG, our strategy was to monitor scalp EEG under all conditions of stimulation, in an attempt to discern stimulation parameters that pose a seizure risk versus those that do not.

Both focal aware and generalized tonic–clonic seizures in response to visual cortical stimulation have been described. An NIH study of a blind participant with 38 intra‐cortical electrodes implanted in the lateral visual cortex for 6 months described a simple focal seizure induced by stimulation [9]. The researchers paid special attention to phosphene descriptions and fluctuations in thresholds and brightness after repeated stimulation and noted both stable phosphenes and unexpected visual experiences described by their participants. After cessation of stimulation of a particular electrode, a focal seizure was observed in which the participant reported a “BB” sized, amorphous, gray‐white phosphene that the participant enjoyed watching while it continued to brighten and enlarge, flickering in colors, eventually covering the contralateral visual field for several minutes. Weeks later, the researchers stimulated that electrode a second time with the same results and elected not to stimulate it again. Scalp EEG was not recorded in that study.

Groups that have implanted cabled “Utah arrays” [16] in nonhuman primates [17] and humans [13] have enabled simultaneous stimulation of the primary visual cortex while recording from arrays implanted in higher‐order visual areas. Chen et al. [17] described both increased and decreased single‐unit spike activity and electrocorticographic (ECoG) changes in response to stimulation. Seizures do not appear to be elicited with subcortical visual or cochlear implant stimulation in healthy subjects, likely due to natural limits on the propagation of activation to the neocortex [18]. This study aims to draw attention to the value of scalp EEG recordings during cortical visual prosthesis testing for real‐time identification of epileptogenic patterns before clinical signs may reveal them.

## 2 Methods

### 2.1 Participants

The first participant was a 53‐year‐old male who was medically, neurologically, and cognitively normal, with a history of myopic retinal degeneration, resulting in 7 years of bare light perception without form vision in the left eye, and enucleation of the right eye following recurrent bilateral corneal inflammation. Since WFMA implantation, the participant has been studied over a 3‐year period. The second participant, a 65‐year‐old male, gradually lost vision as a result of an automobile accident at age 17; initial characterization and mapping of the elicited phosphenes are ongoing in preparation for camera‐driven stimulation. This research was conducted in accordance with the principles embodied in the Declaration of Helsinki and in accordance with local statutory requirements. It was approved by the Rush University Institutional Review Board under ORA protocol #20041305‐IRB02.

### 2.2 Implantation and Testing

**FIGURE 1:**
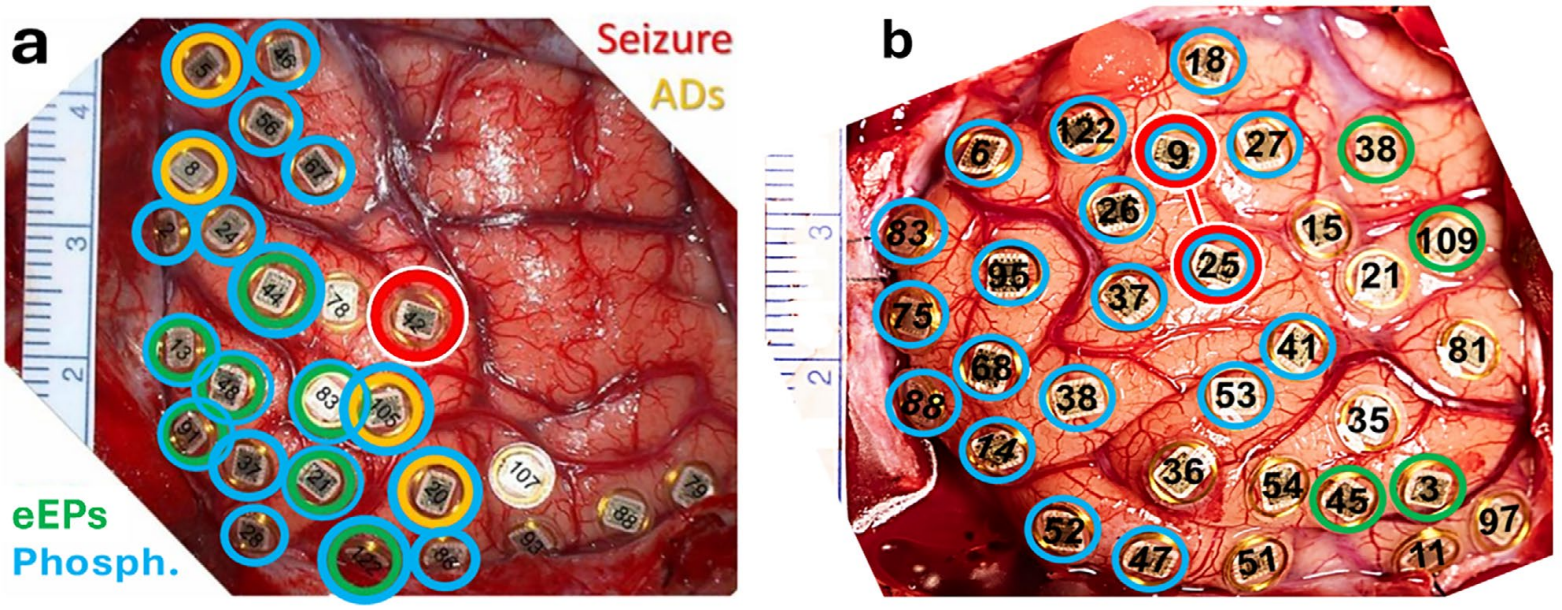
Photographs of numbered WFMAs in situ. Blue circles indicate WFMAs that generated phosphenes. Green circles indicate WFMAs that evoked eEPs. Yellow circles indicate WFMAs that elicited after‐discharges, and the red circles indicate WFMAs that elicited a seizure. WFMAs without circles were unresponsive to stimulation. (a) Participant 1: Ruler is near the midline. (b) Participant 2: Yellow dashed line parallels the midline. Implants extend farther in the temoral direction. eEPs will be fully characterized over the next 2 years. [Color figure can be viewed at wileyonlinelibrary.com]

**FIGURE 2:**
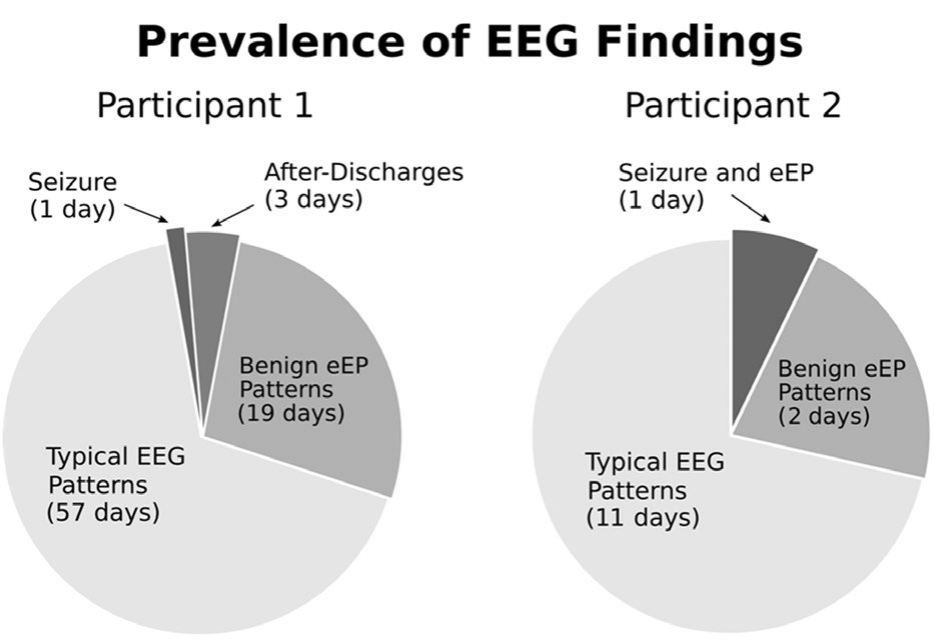
(Left) Participant 1's proportions of days in which different EEG scalp patterns were recorded. (Right) Although not studied for as long, Participant 2 experienced similar proportions of activity.

The first participant underwent preoperative cerebral imaging followed by implantation during a 5‐h right occipital craniotomy, during which 25 WFMAs were implanted in the dorsolateral occipital cortex; each containing eight 1.3 mm and eight 1.6‐mm‐long intracortical stimulating electrodes, resulting in 400 independent electrodes (Figure 1a). The second participant received 32 intracortical WFMAs, totaling 512 electrodes (Figure 1b). After a prescribed 1‐month recovery period, Participant 1 underwent 3 years of testing to determine the stability of threshold current levels and to map phosphene locations and characteristics. This resulted in 274 h of testing with continuous EEG monitoring across 80 nonconsecutive sessions. An additional 56 h of EEG were recorded spanning 15 nonconsecutive days for Participant 2. As described below, three categories of signals were observed: (1) asynchronous normal occipital EEG patterns, EMG artifacts, and telemetry interference, (2) electrically evoked potentials (eEPs) to stimulation, and (3) induced after‐discharges and seizure activity (Figure 2).

### 2.3 Wireless Floating Microelectrode Arrays

Detailed technical descriptions of the 5 mm diameter WFMAs have been published [19, 20, 21, 22]. Briefly, the WFMAs are transcutaneously powered and use near‐field magnetic coupling for communication via an extracorporeal 9‐cm diameter coil. This coil operates at a carrier frequency of 4.6 MHz, using frequency‐shift‐keyed modulation at a data rate of 1.125 Mbits/s. A reverse telemetry channel enables two‐way communication between the WFMAs and the external telemetry system by transmitting commands through a low‐frequency (145 kHz) data subcarrier. The WFMAs receive power induced from the external coil (4.6 MHz), eliminating the need for implanted batteries. Our current WFMAs do not have the ability to record and transmit intracranial EEG, necessitating the use of scalp EEG recordings. The Intracortical Visual Prosthesis (ICVP) configuration for the two participants employed various stimulation sequences using cathode‐first biphasic pulses (100–400 μs phase duration) presented between 70 and 200 Hz utilizing single electrodes, electrode pairs, quads, and complete WFMAs. These direct stimulus trains could last as long as 30 s; longer trains were allowed with camera‐driven stimulation.

### 2.4 Scalp EEG Recordings

Two‐channel occipital EEG scalp recordings were derived from the international 10–20 EEG system (O1‐Fz, O2‐Fz, ground: Cz). EEG channels O1 and O2 were selected for their proximity to the posterior lateral visual cortex, enabling direct comparison of signals between the stimulated and unstimulated visual cortices. These recordings were monitored during threshold determinations, phosphene mapping, direct intracortical stimulation, and camera‐driven sessions. The two records were amplified and digitized at 500 Hz each using a Brain Vision Products V‐Amp 16‐channel amplifier (Garner, North Carolina, USA) and displayed using open‐source 64‐bit OpenViBE v3.4.0. Typical recordings were filtered at 1–30 Hz using a Butterworth fourth‐order bandpass filter to minimize waveform drift from baseline and attenuate high‐frequency artifacts from the nearby telemetry field. Each stimulating electrode had its own threshold level, so stimulus amplitudes for multielectrode stimulation were set at a common multiple of these thresholds (e.g., 1.8 × threshold current) rather than at a common current level. To interrupt stimulation in case of suspected epileptic activity, the participants wore a stop‐stimulation button around their neck, while staff also had similar buttons at hand.

### 2.5 Threshold Determination and Camera Interface

Threshold levels for each of 2030 electrode/parameter combinations were measured up to 55 times across up to 1022 days. Current was varied within 0.7–77.5 μA, holding other parameters constant. Typical stimulation parameters included 200 Hz, 400 ms trains, 200 μs cathodic phases in cathodic‐first pulses, and 62 μs delays between successive electrode onsets. The 63% hit‐rate threshold was determined assuming a Weibull distribution.

Each camera image was processed in its native resolution of 1280 × 720 pixels. The location of the region of interest for each electrode group was determined according to their phosphene location and size within the camera field. Lists of electrode groups to activate to convey the camera image were updated every 40–50 ms, yielding a frame rate of approximately 20 Hz.

### 2.6 Six Goals of Stimulation Studies

Briefly, there were six goals of stimulation: (1) Periodically assess the health of the electrode/tissue interface, to ensure that there was no damage to the tissue or to the surface of the electrodes [19]. (2) Determine the stability of thresholds required to detect a phosphene on 63% of stimulations. (3) Determine the location in the visual field and subjective characteristics of the elicited phosphenes. (4) Determine the degree to which closely spaced phosphenes interact with each other. (5) Stimulate small groups of electrodes nearly simultaneously in order to reduce the peak stimulation current density in tissue. And (6) Stimulate electrodes according to the subsampled video stream from a camera attached to the participant's glasses if the pixel brightness at the corresponding image location exceeded a specified level.

## 3 Results

During Participant 1's initial 80 nonconsecutive days of monitoring, several different scalp EEG patterns were observed, including typical normal EEG patterns (57 days), benign eEPs and blink‐induced artifacts (261 on 19 days), abnormal seizure‐like activity (3 days) and a single seizure (1 day) (Figure 2, left). During Participant 2's 14 nonconsecutive days of monitoring, typical EEG patterns were observed (11 days), along with benign eEPs (2 days) and seizure‐associated activity (1 day) (Figure 2, right). The characteristics of various types of scalp‐recorded EEG phenomena are described below.

### 3.1 Radio‐Frequency Artifacts

**FIGURE 3:**
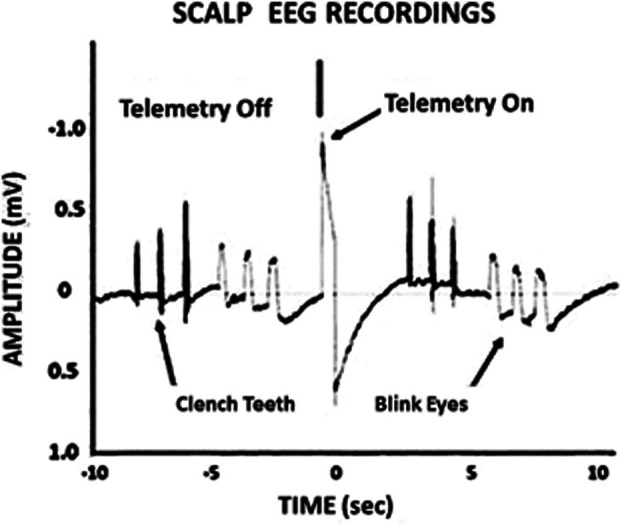
Low‐amplitude display comparing voluntarily emitted myographic artifacts and induced electronic signal interference recorded from a healthy subject. Note the brief baseline shifts and high‐frequency artifacts when telemetry was turned on. The low vertical display of these records does not reveal the high‐frequency artifact that occasionally distorts normal EEG patterns.

Aside from the easily recognized EEG artifacts from muscle activity and eye movements, high‐frequency artifacts from the EEG telemetry system coil placed directly above the O_2_ electrode were occasionally observed in the recordings. These artifacts could be minimized by ensuring that scalp electrode impedances were low (< 5 kΩ) and that EEG wires were arranged perpendicular to the induced field, with bandpass filters set from 1 to 30 Hz (Figure 3). Occasionally, the high‐frequency cut‐off filter frequency was raised to investigate the nature of high‐frequency waveform interference. EEG channels O1 and O2 were selected for their anatomical proximity to the visual cortex, allowing for the direct comparison of analogous ipsilateral and contralateral signals over the WFMAs.

### 3.2 Electrically Evoked Potentials (eEPs)

**FIGURE 4:**
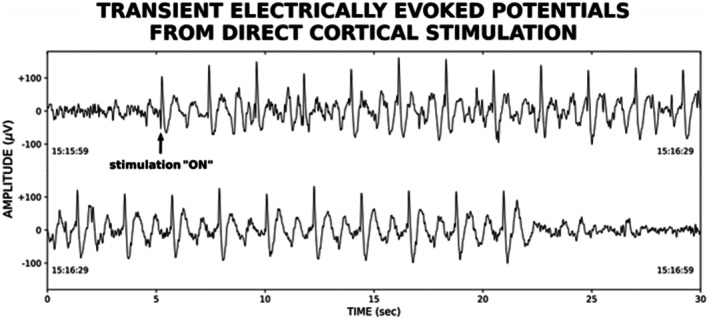
Participant 1's occipital scalp EEG recordings from O_2_ during 22 trains of activations of 14 WFMAs eliciting central phosphenes (200 μs phase duration, presented at 160 Hz, with 45 μA) with a stimulation “ON” at 15:16:04.21. Each stimulus was followed by a transient eEP consisting of a discrete pattern of high‐amplitude responses primarily over the right hemisphere. The resting EEG immediately returned when the stimulus train was terminated. Numbers here and in subsequent figures indicate local time.

During the initial phase of testing, repeated stimulation of single electrodes to determine phosphene thresholds and their location in the visual field rarely elicited waveforms detectable in the raw EEG. Later in the study, when higher levels of stimulation were used, or multiple electrodes were stimulated nearly simultaneously, eEPs (also referred to as eVEPs by [20]) were frequently observed in the raw scalp recordings. Benign eEPs were observed 261 times during direct cortical stimulation or camera‐based stimulation. These eEPs resemble normal activation of the visual cortex, similar to visual evoked potentials in sighted individuals. Figure 4 depicts an example of transient eEPs with complex, repetitive waveforms evoked (response phase being tightly locked to the stimulus) by nearly simultaneous pulses from 56 electrodes across 14 different WFMAs during a 460‐s stimulation train for Participant 1. The train consisted of 1.1‐s bursts of 160 Hz biphasic pulses (cathodic phase duration 200 μs, 45 μA) at 2‐s intervals. Additionally, eEP waveforms from Figure 4 were analyzed using a one‐sample t‐test, comparing their parameters to those of a conventional scalp VEP waveform (P100, N140). Although the waveforms appeared qualitatively similar to traditional VEPs, their mean P‐N interval of 53 ms was significantly longer than the anticipated 40 ms (t = 28.59, df = 21, *p* < 0.0001), indicating that the underlying cortical activity did not match that of a typical VEP.

**FIGURE 5:**
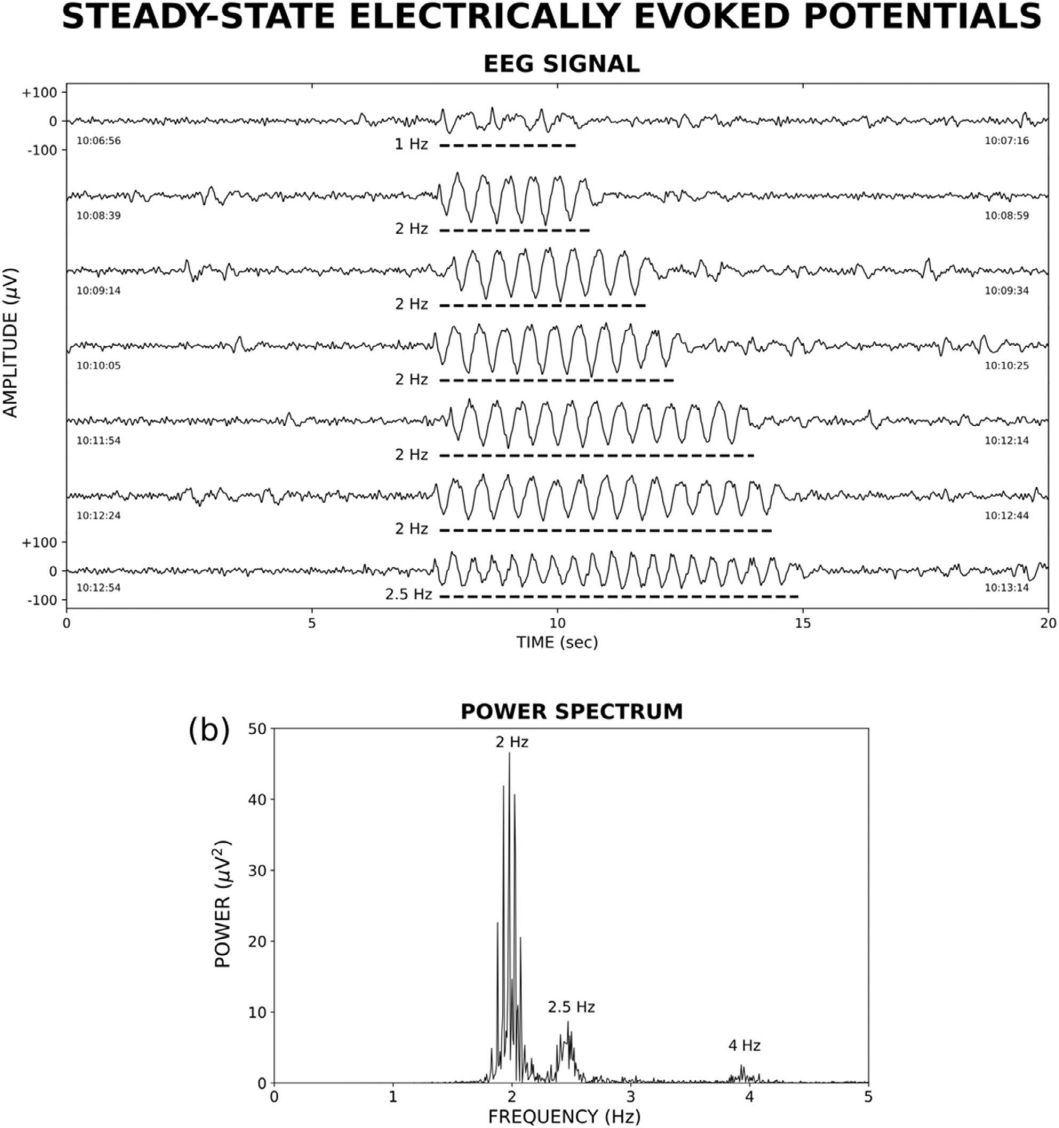
(a) Steady‐state eEPs were evoked by direct rapid stimulation trains to the six WFMAs selected for future camera use (phase duration 200 μs, 200 Hz at 35 μA). Six WFMAs were simultaneously activated with train lengths ranging from 3 to 7 s. The duty cycles were the following: 400 ms on / 600 ms off, 200 ms on / 300 ms off, and 200 ms on / 200 ms off. Dashed lines: Train duration with corresponding repetition rate (Hz). The resting EEG immediately returned when the stimuli were terminated, but subjective phosphene persistence lasted for as long as 14 s. The top trace evoked individual waveforms, with subsequent traces showing the transition from transient to steady‐state responses. (b) Power spectrum calculated across all the tracings combined in Figure 5a revealed increased power at the fundamental frequencies of 2 Hz and 2.5 Hz.

When repetition rates were increased, the transient eEPs in the scalp EEG started to overlap, producing a steady‐state sinusoidal‐like waveform that began with the first stimulus and ceased when the train of stimuli was halted. Figure 5a illustrates steady‐state eEPs evoked by more rapid trains of pulses presented to pairs of electrodes in multiple WFMAs eliciting white phosphenes in the central visual field, ranging from 3 to 14 bursts of stimulation (200 ms on, 200 ms off, 200 μs phase duration presented at 200 Hz at 35 μA). These eEPs followed the repetition rate and began and ended with the stimuli. In some instances below, the participant reported the persistence of the visual percept after the stimulation and response had ended. Manipulation of stimulus intensity indicated that at high stimulus intensity, these waveforms could retain some of the stimulus artifact seen in the transient responses in Figure 4, along with physiologic responses.

### 3.3 After‐Discharges

**FIGURE 6:**
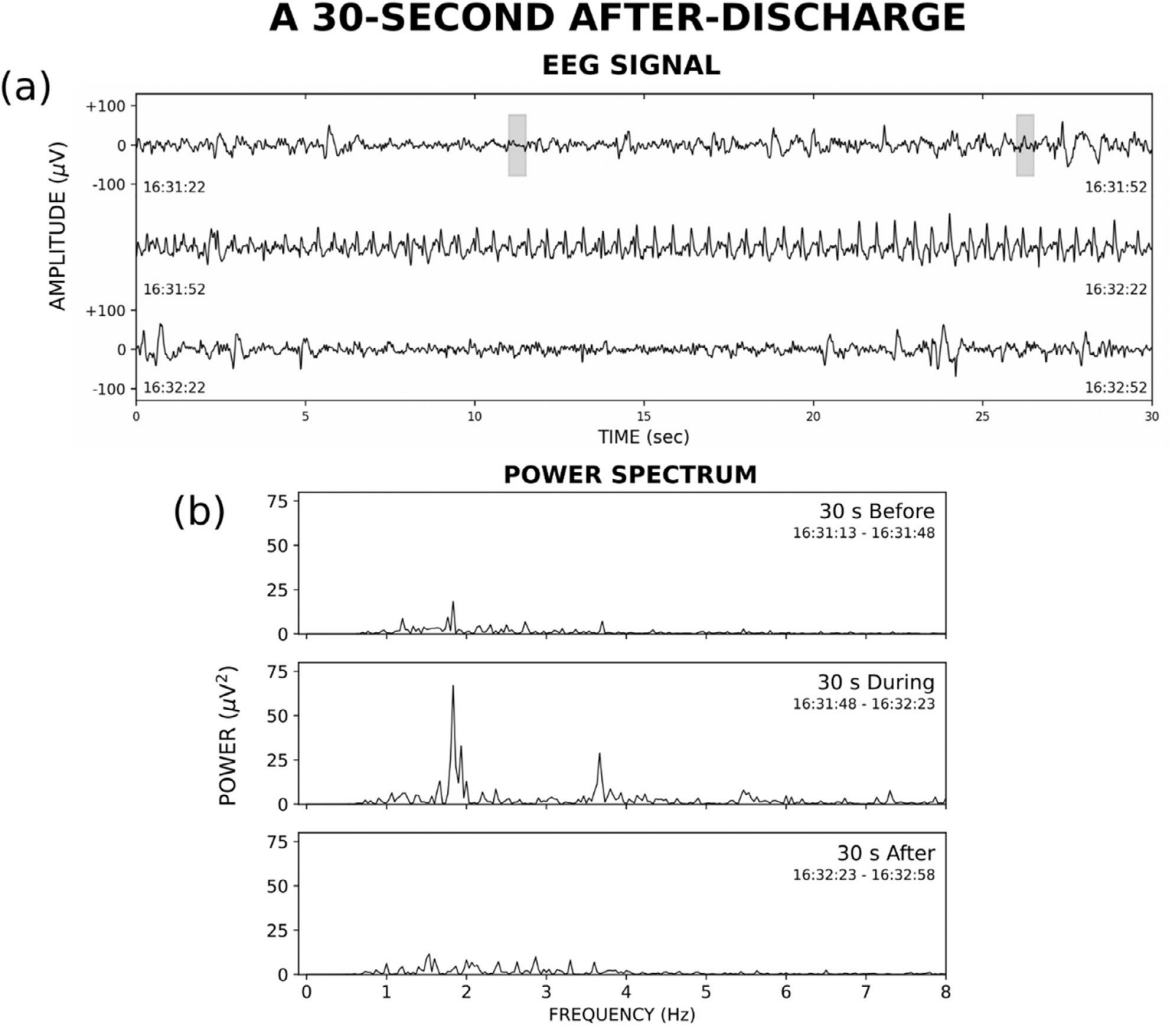
(a) An example of successive epochs of a 30‐s after‐discharge recorded from the scalp over the WFMAs (electrode O_2_). After direct cortical stimulation at 16:31:33 (WFMA 8) and 16:31:48 (WFMA 20, 200 Hz, 200 μs phase width, 1‐s train duration at 60 μA) (gray boxes), a spike‐and‐wave pattern with persistence spontaneously ended at 16:32:23. (b) Fourier transform for a 30‐s after‐discharge showing dominant frequencies during the 30‐s intervals before, during, and after the after‐discharges. The lower power spectrum highlights a return to resting state following the 30‐s after‐discharge.

**FIGURE 7:**
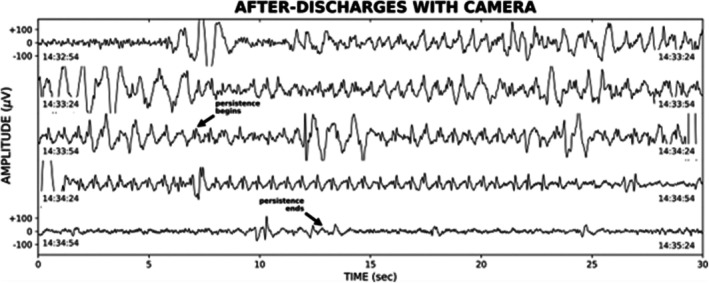
Participant 1's continuous 150 s of EEG while examining a vertical white bar during camera‐controlled input. After‐discharges appear and continue until 15 s before the persistence of the phosphenes ends.

We observed seven instances of after‐discharges: one during camera‐driven stimulation and six following direct cortical stimulation. Three after‐discharges were recorded prior to the single, late‐afternoon seizure elicited in Participant 1. Like persistence, after‐discharges were elicited by the stimulus but could continue independently. These events typically lasted 2–3 s but occasionally continued for several minutes before spontaneously resolving. After‐discharges often decreased in amplitude and slowed in frequency before self‐terminating. If after‐discharges lasted more than 2–3 s and were accompanied by visual persistence, there was an increased risk of progression into a seizure. After‐discharges associated with camera stimulation consisted of slow, 1–2 Hz high voltage (400 μV) oscillations or variable spike‐and‐wave patterns of lower voltage (Figure 7). Unlike eEPs, which are closely related to the evoking stimulation and do not elicit seizures, induced after‐discharges and seizures follow the stimulation and may gradually increase in amplitude and frequency (Figure 6a).

After‐discharges (and eEPs) can also occur during camera‐controlled stimulation (Figure 7). This is particularly concerning because we did not have the capability of recording scalp EEGs during ambulation, and the participant might not be aware of these events and discontinue stimulation using the stop button. The effects of after‐discharges may be cumulative, as observed in our case, suggesting that it would be prudent to terminate testing for at least the remainder of the day.

### 3.4 Focal Aware Seizures

Cortical hyperexcitability was observed in two instances of electrographic seizure activity. Participant 1 experienced clear clinical manifestations consistent with a focal aware seizure. Participant 2 experienced only transient visual symptoms, without additional clinical manifestations; this was not recognized as a seizure until it was reviewed several days later.

**FIGURE 8:**
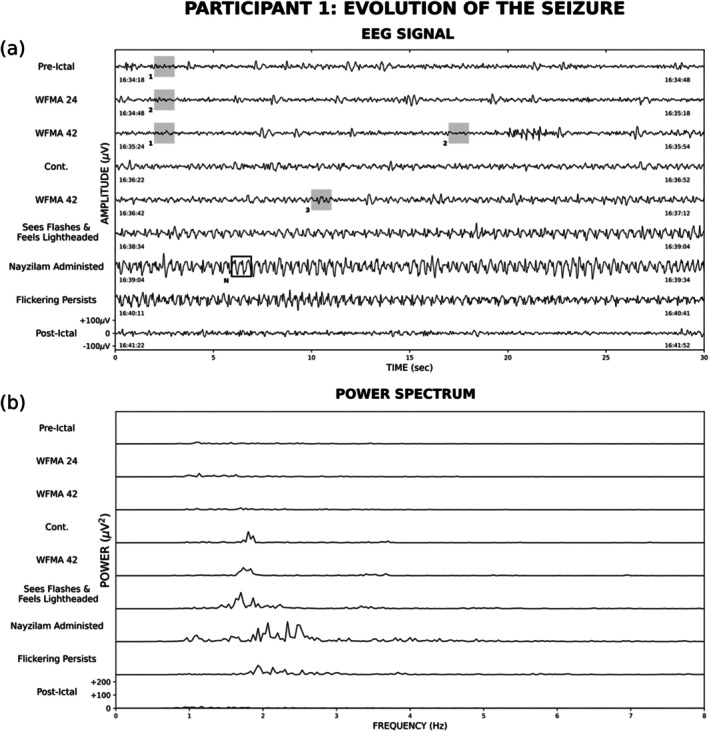
(a) Selected 30‐s segments of the O2 EEG showing the evolution of Participant 1's seizure. Gray boxes indicate the 1‐s duration stimuli as described in the text. Segments include the preictal, ictal, and postictal EEG after the nasal administration of antiseizure medication to prevent generalization. The brief transients in the top four traces are eye blinks from the Fz reference electrode. WFMA 24 elicited phosphenes twice but did not elicit a response in the EEG. Conversely, WFMA 42 did not elicit a phosphene. The second activation of WFMA 42 elicited brief after‐discharges, but the onset of the seizure was not recognized until the third stimulation of WFMA 42, less than a minute later. Numbers indicate how many times each stimulus was presented. Black box (N) indicates the administration of Nayzilam nasal spray. (b) The Fourier transform for the evolution of the seizure demonstrates an increase in rhythmic low‐frequency activity at 1–4 Hz, commonly observed in ictal phases. Timestamp 16:41:24 shows a reduction in low‐frequency power, indicating the seizure was resolving, aligning with a postictal state.

During one session, we re‐examined WFMAs that had previously not elicited phosphenes. We stimulated all 16 electrodes on individual WFMAs nearly simultaneously (200 μs pulse width, 1‐s train, 200 Hz, 60 μA, with a 62 μs delay between electrodes) to elicit multiple phosphenes. Repeated stimulation of WFMA 24 elicited a golf‐ball‐sized phosphene, whereas WFMA 42 did not elicit a phosphene, but instead elicited a seizure (Figure 8). The seizure began 3 s after stimulation with evolving 2 Hz after‐discharges, which increased in amplitude and frequency, evolving into a continuous spike‐and‐wave pattern. Subsequently, the participant reported seeing diffuse flashes, followed by bilateral autonomic signs and symptoms, which resolved 1.5 min after the intranasal administration of Nayzilam as an abortive antiseizure medication. The participant did not experience any extended symptoms from the seizure. The semiology was consistent with a focal aware seizure originating in the right occipital cortex, without generalization. It remains unclear whether the synchronous stimulation presented before the second stimulation of WFMA 42 might have contributed to the seizure onset. Notably, this seizure occurred only a few minutes after the after‐discharges depicted in Figure 6 (as indicated by the local 24‐h clock time). This was the only seizure observed in Participant 1 in the study. It should be noted that for the first time, the participant reported poor sleep quality on the preceding night, rating the quality as 4 on a subjective 1–10 scale, compared to his usual score of 7/10. Our protocol required a 2‐month delay in testing following a seizure.

**FIGURE 9:**
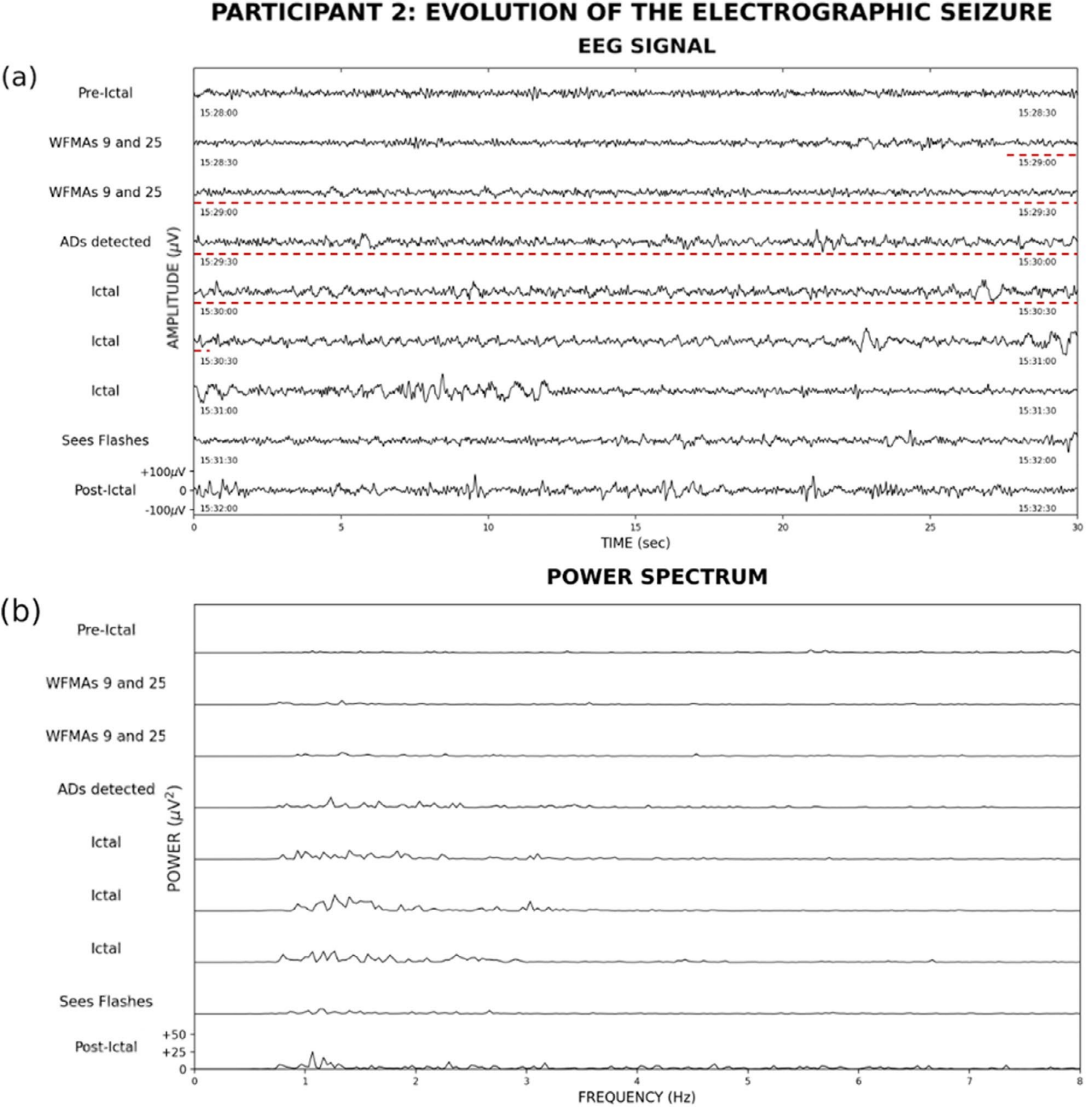
(a) Consecutive 30‐s recordings from the O2‐Fz scalp channel during Participant 2's adverse event. Stimulation parameters were set to 200 Hz frequency, 200 μs phase duration, 29 μA current, switching back and forth between WFMAs 9 and 25 (red dashed line). Segments illustrate the progression of the seizure, highlighting after‐discharges (ADs) detected prior to the onset of sustained (> 1 min) ictal activity. Participant 2 reported perceiving a flash of light approximately 1 min after stimulus termination, coinciding with the transition of waveforms into a postictal state characterized by a return to lower amplitude fluctuations. (b) The Fourier transform illustrates subtle changes in frequency throughout the adverse event, consistent with the reduced severity of the seizure event. [Color figure can be viewed at wileyonlinelibrary.com]

For our second participant, out of caution, we initially continued to record scalp EEG under all conditions. During one of the testing conditions aimed at determining the relative location of phosphenes, where stimulation was toggled between arrays 9 and 25 at a current of 29 μA, the second participant exhibited after‐discharges that evolved into a brief seizure evident in the EEG (Figure 9). Following immediate termination of stimulation, the second participant experienced transient visual phenomena reported as a flashing light without other apparent clinical manifestations of a seizure, consistent with a focal aware seizure. Administration of an antiseizure medication was not indicated, given the absence of apparent clinical manifestation and prompt return to baseline in the EEG following stimulus offset. Although restrictions on high‐intensity or multi‐electrode stimulation were followed to mitigate the risk of future seizures, a focal aware seizure still occurred but passed undetected in our second participant. However, both the clinical severity and duration were reduced. Scalp EEG monitoring facilitated early detection of abnormal activity, enabling prompt stimulus termination and preventing escalation of seizure severity when indicated.

## 4 Discussion

This study demonstrates that real‐time seizure detection using scalp EEG during intracortical visual prosthesis testing can provide an early warning of a seizure caused by entrainment of the visual cortex. This approach is particularly important, as the implanted wireless floating microelectrode arrays used in our study lack the capability to record intracranial EEG signals. Under specific conditions—such as the nearly simultaneous activation of several intracortical electrodes, stimulation intensities decidedly above threshold, or long durations of stimulation—there is an increased risk of seizures. In these instances, scalp EEG monitoring can have an important role in ensuring participants' safety. Conversely, determining phosphene thresholds or mapping locations for single electrodes does not usually produce a signal in the EEG and does not appear to increase the likelihood of seizures, suggesting that not all testing may require EEG monitoring. Nonetheless, some electrodes may not be in cortical areas dedicated to vision and will not yield phosphenes. High‐intensity stimulation of such electrodes poses a risk of inducing seizure activity without eliciting visual sensations.

In addition to detecting seizures and after‐discharges, EEG recording revealed numerous normal and natural eEPs in response to stimulation, reminiscent of transient and steady‐state VEPs from subjects with intact visual pathways [23, 24, 25]. These eEPs usually appear when a stimulus train is initiated and cease when the train of stimulation ends. However, in some instances, the phosphenes would gradually fade during stimulation, but the eEP continued until the stimulus ended. This dissociation between the duration of the phosphene and the eEP suggests that some form of inhibition may develop at higher visual areas, which are more closely associated with conscious perception. Conversely, the perceptive persistence of phosphenes unaccompanied by EEG phenomena in our limited montage may imply conscious, uninhibited physiologic processing underlying visual perception in higher visual areas [26] or the frontal lobe [27]. Chen et al. [26] also noted that V1 stimulation elicited transient far‐field eEPs recorded in primate V4, which might serve as a proxy for a participant's subjective response during preliminary threshold testing. If validated, this might significantly reduce the length of threshold determinations. Poort et al. [28] noted that early figure‐ground separation is enhanced by interactions between primate V1 and V4.

It is likely that such eEPs and seizure activity could be recorded more easily from intracortical electrodes rather than with scalp recordings. It is important to note that we observed eEPs and after‐discharges not only during experimentally controlled direct cortical stimulation but also with external camera‐controlled stimulation.

Natural human vision is spontaneous and appears to involve an effortless “bottom up” signal processing sequence. In contrast, visual prosthetic vision has been described as a qualitatively different top‐down “contemplative perception” [29], which is an effortful attempt to decide what the relatively sparse distribution of artificial phosphenes in the subjective visual field may represent. This may be due to the practice of using relatively high above‐threshold stimulation to obtain reliable phosphene mapping results, which may disrupt normal neuronal processes. In our experience, such high stimulation currents or long durations may contribute to after‐discharges. It appears that such stimulation may be cumulative, in that our data indicate that in rare cases no activity was recorded after a first presentation of a stimulus sequence, but that the second time the stimulus was administered, it elicited after‐discharges or even a seizure [30]. To create a more natural form of vision, it may be necessary to decrease the stimulus strength to gently modulate, rather than overwhelm ongoing innate visual processing activity; it is likely, however, that this will require more and denser electrode arrays.

As visual prosthetic systems evolve, they ought to utilize local intracortical stimulation and widespread ECoG recordings simultaneously, as has been done with Utah arrays in nonhuman primates. Chen et al. [17, 26] recorded single‐unit activity after stimulation and noted that stimulation either increased or decreased the firing rates. It seems likely that alteration of a stimulus or presenting specific stimuli may become useful for preventing after‐discharges from escalating to seizures. Phogat and Parmananda [31] found that primate V1 and V4 are strongly connected and play a key role in figure‐ground separation. This raises the possibility that V4 stimulation may subtly modulate visual perceptions.

Several precautions can be taken in order to minimize the risk of seizures during intracortical visual prosthesis testing: (a) exclude prospective participants with a history of seizures; (b) perform an extended clinical EEG in the initial work‐up; (c) consider prescribing prophylactic oral antiseizure medication; (d) train staff working with participants in acute seizure management; (e) include scalp EEG or ECoG to safely assess the effects of electrical stimulation during preliminary investigations, particularly when an increasing number of electrodes will be stimulated simultaneously, at high stimulus currents, when rates of stimulation or long duration trains are presented, and during initial camera‐driven stimulation; (f) avoid stimulating large numbers of electrodes simultaneously; (g) consider using temporal interleaving of nearby electrodes [14]; (h) have an abortive antiseizure medication readily available during all sessions; and (i) routinely assess sleep quality because sleep‐deprivation is a routine clinical manipulation used to provoke seizures in epilepsy patients [32, 33] and may be a contributing factor that alters cortical excitability during electrode stimulation [34]. Participant 1's seizure occurred following a reported poor night of sleep, combined with an unusual number of after‐discharges.

A review of our overall experience during this period revealed that spike‐and‐wave after‐discharges and the seizures in two participants were triggered by repeated stimulation of all electrodes in an array at moderate intensities and during camera‐driven stimulation (Figures 6, 7, 8). The power spectra from eEPs were less variable than those recorded during seizures. After the first year of continuous recordings with our first participant, we observed that single or paired electrode threshold studies and spatial mapping studies, which comprise the majority of study time, did not produce any stimulus‐related signals in the EEG and therefore would not have required EEG monitoring (Figure 2). However, when multiple electrodes were stimulated or higher intensity stimulation was employed, it was prudent to record EEG.

## 5 Limitations

This study involved recordings from only two participants, which limits generalization. Other participants may have a different propensity toward stimulation‐induced seizures, which should be assessed early on with EEG recordings. Our first participant did not tolerate prophylactic oral antiseizure medication but responded immediately to an abortive antiseizure medication intranasally. Another limitation was that only two active EEG channels, O1 and O2, were recorded, preventing observation of potentially relevant phenomena from other parts of the scalp. Due to occasional high‐frequency interference from the telemetry coil, which was placed directly over the O_2_ electrode, we used a slightly lower high‐frequency cut‐off than customary, precluding the ability to examine higher frequency EEG bands. We also did not have the capability to signal‐average responses to identify low‐voltage eEPs to identify possible stimulation artifacts volume conducted from the intracortical electrodes. Finally, our WFMAs have been studied for only 3 years. We have noted a few and only modest increases in thresholds over this period, suggesting that electrode surface breakdown, encapsulation, or neural changes from stimulation, reported in primate visual cortex studies [25], do not significantly affect the prospects of long‐term WFMA use. It is the opinion of our neurosurgeons that the implanted WFMAs should permanently remain in place unless excision is requested by the participants before the end of the study. Our current observations are likely to be limited to a 5‐year period dictated by funding constraints.

We have largely addressed the safe and reliable management of direct cortical stimulation to map the spatial and perceptual characteristics of phosphenes necessary to implement a camera interface. Once a camera‐based visual interface is implemented, the next critical step will be to safely modulate the somewhat unpredictable nature of the camera‐driven stimulation, particularly as it involves the interactions of a large number of phosphenes. Camera‐driven stimulation poses a particular concern because the participant controls the input and may not be aware of adverse events. If groups of nearby phosphenes are activated, it might be prudent to activate them with lower currents to reduce the build‐up of inhibition [35] or dither their activation patterns laterally to create artificial micro‐saccades to decrease the fading that has been associated with stabilized cortical stimulation [36]. Although our participants easily specified the location of phosphenes in their subjective visual field (presumably using the superior “where” pathway), a notable limitation of our study (and others) is that we did not discern any activation of the inferior “what” pathway [37]. Whether this is due to the relative paucity of electrodes relative to normal afferents, or our failure to optimally activate, rather than disrupt, existing cortical networks, remains a challenge for future investigations in this field.

## 6 Conclusions

Experience with continuous recording of scalp EEG during visual prosthesis testing revealed a variety of electrophysiologic phenomena that are not yet completely characterized. Some of the stimulus‐related potentials may have a specific role in advancing our understanding of the neurophysiological underpinnings of artificial vision. After becoming familiar with a participant's EEG during extensive investigations, we observed that EEG monitoring is not necessary for some stimulation conditions, such as threshold determination and localization of phosphenes from single or paired electrodes. However, for higher‐risk stimulation conditions, optimized limited EEG montages as utilized in this study, combined with the availability of abortive antiseizure medication, can aid in the real‐time identification of seizure onsets and reduce the associated risk to participants. We look forward to a future where prosthetic implants integrate the dual functionality of stimulation and intracranial ECoG recordings as a feature of the telemetry system, obviating the need for scalp EEG during visual prosthesis testing.

## Author Contributions

**Katarina Stephan:** data analysis, manuscript writing. **Philip R. Troyk:** designed and led prosthesis development. **Gislin Dagnelie:** reviewed EEG, manuscript writing. **Stuart Cogan:** developed implants, liaison with FDA. **Michael P. Barry:** created stimulation software, conducted participant sessions. **Patricia Grant:** supervised participants, manuscript writing. **Frank Lane:** supervised enrollment and care of participants. **Janet P. Szlyk:** built laboratory, managed participants. **Maggie McNulty:** evaluated seizures. **Wim van Drongelen:** devised seizure detection parameters. **Tuan H. Pham:** developed seizure analysis software. **Kelsey L. Stipp:** screened and managed participants. **Meesa Royster:** screened and managed participants. **Vernon L. Towle:** developed EEG protocol and recorded EEG, participant safety office.

## Conflicts of Interest

The authors declare no conflicts of interest.
